# Reference Values for Five-Repetition Chair Stand Test Among Middle-Aged and Elderly Community-Dwelling Chinese Adults

**DOI:** 10.3389/fmed.2021.659107

**Published:** 2021-04-21

**Authors:** Shan-Yan Gao, Yang Xia, Qi-Jun Wu, Qing Chang, Yu-Hong Zhao

**Affiliations:** ^1^Clinical Research Center, Shengjing Hospital of China Medical University, Shenyang, China; ^2^Department of Clinical Epidemiology, Shengjing Hospital of China Medical University, Shenyang, China; ^3^Department of Graduate Medical Education, Shengjing Hospital of China Medical University, Shenyang, China

**Keywords:** community-dwelling, five-repetition chair stand test, older adult, physical function, reference value

## Abstract

**Background:** Previous studies on the five-repetition chair stand test (CS-5) are limited by the representativeness of the sample or the lack of reference equations for CS-5. Defined reference values for CS-5 in a large population are not available for middle-aged and elderly Chinese adults.

**Objective:** We established age- and sex-stratified reference values for CS-5 times in a large population in China, and to investigate the associations between demographic and anthropometric factors and CS-5 times.

**Methods:** Analysis of data from the national baseline survey of the China Health and Retirement Longitudinal Study, a nationally representative longitudinal survey that includes 450 urban communities and rural villages within 28 provinces, municipalities, and autonomous regions of China.

**Results:** Twelve thousand six hundred five of seventeen thousand seven hundred eight participants were included for the reference value analyses. Twelve thousand three hundred out of seventeen thousand seven hundred eight participants were included for the risk factor analyses. Of 12,605 participants, the mean CS-5 time was 10.13 s (SD, 3.32) in men and 11.03 s (SD, 3.54) in women aged 40+ year. The CS-5 times were shorter in men than women of all age categories (*P* < 0.001). The cut-off points ranged from 5.36 to 9.98 s and from 6.48 to 10.29 s in men and women, respectively. Mean velocity was higher in men than in women (*P* < 0.001). Age, waist circumference, living in a rural village, and having chronic diseases were positively associated with CS-5 time, whereas male, handgrip strength, currently married, income, and current or ex-drinker were negatively associated with CS-5 time in this population (all *P* < 0.001).

**Conclusions:** The comprehensive normative values for CS-5 are essential for enabling clinicians to better evaluate functional performance, determine the appropriate interventional strategy, and promote healthy aging of older adults.

## Introduction

Aging of the population in China is growing and becoming a very serious problem. In 2010, about 111 million (8.2% of the total population) older adults were identified in China and about 19.3 million were >80 years old. Due to the effect of the one-child policy and the increase in life expectancy, it is estimated that there will be 400 million older adults in 2050 (26.9% of the total population), 150 million of whom will be >80 years old (1). Declining muscle function due to losses in muscle mass and muscle strength is considered a hallmark of the aging process (2). Recent studies have shown that higher muscle mass and muscle strength are conversely associated with lower morbidity and all-cause mortality in older adults (3, 4). Early clinical detection of functional decline allows for an intervention and prevents a further decline in physical function and independence.

The chair stand test is a widely implementable test used to assess physical functioning and lower body muscular strength and endurance, particularly among older adults. The chair stand test requires little training to administer and uses simple equipment (conventional chair and a stopwatch), such as the five-repetition chair stand test (CS-5), 30-s chair stand test, and 1-min sit-to-stand test (5). Of these, the CS-5 captures a subject standing from a seated position five times. It may be used as an individual measure or as part of the Short Physical Performance Battery to assess physical functioning (6). A poor performance time on the CS-5 has been used as a predictor of falls and decline in activities of daily living in a wide range of functional outcomes, including chronic obstructive pulmonary disease (7), stroke (8), and musculoskeletal symptoms (9). The CS-5 demonstrates clinical significance for diagnosing limited mobility, aiding in the prognosis, and comparing groups or evaluating the effectiveness of interventions on physical functioning (10).

A comparison with age- and sex-matched normative reference data generated from a large population undergoing the chair stand test is required for use in clinical practice. Several studies [four studies for CS-5 (11–14)], three studies for the 30-s chair stand test (15–17), and one study for the 1-min chair stand test (18) have reported reference values for the chair stand test in the past decade. A previous meta-analysis reported that the reference value for the CS-5 was 8.50 s [95% confidence interval (CI): 7.93–9.07], and the reference value for the 30-s chair stand test was 17.26 times (95% CI: 15.98–18.55) in healthy Japanese older adults (19). However, existing datasets for the CS-5 are limited by the representativeness of the sample or the lack of reference equations for CS-5 and details of the chair characteristics. Defined reference values for CS-5 in a large population are not available for a large aging population like that in China.

Thus, the aim of the present study was to establish age- and sex-stratified reference values for CS-5 times in a large soon-to-be old and older adult population in China, and to investigate the associations between demographic and anthropometric factors and CS-5 times.

## Materials and Methods

### Study Population

The China Health and Retirement Longitudinal Study (CHARLS) is a nationally representative longitudinal survey that includes 450 urban communities and rural villages within 28 provinces, municipalities, and autonomous regions of China. The design and data collection of this cohort study has been described previously (20). The survey included three waves, such as the baseline (W1) 2011–2012 survey, the second wave (W2) 2013–2014 survey, and the third wave (W3) 2015–2016 survey.

This cross-sectional study used data from the CHARLS national baseline survey conducted between June 2011 and March 2012 and included 17,708 participants. We excluded participants who did not provide information on CS-5 time (*n* = 4,802), and participants whose age was <40 years (*n* = 50). We also deleted the top and bottom 1% values on the CS-5 time (*n* = 251). Therefore, 12,605 participants were eligible for reference values analyses (Supplementary Figure 1). Furthermore, we excluded participants who did not provide information on body mass index (BMI) (*n* = 109), smoking status (*n* = 9), waist circumference (*n* = 18), or handgrip strength (*n* = 169). Thus, 12,300 participants were eligible for risk factor analyses (Supplementary Figure 1). This study protocol was approved by the Ethical Committee of Peking University. Written informed consent was obtained from all participants.

### Measurement of CS-5

A CS-5 pre-test was performed on a standardized armless chair (0.47 m height) using a handheld stopwatch. The back of the chair was stabilized against a wall. Participants were asked to fold their arms across their chest (i.e., armrests were not used) and stand up from the chair. If the pre-test was successful, the participants were asked to perform five chair stands as quickly as possible without using their hands to push up from the chair. They were timed (in sec) from the initial sitting position to the final standing position on the fifth stand. The CS-5 mean velocity (in m·s^−1^) was calculated as the vertical distance (m) covered by the center of mass divided by the mean time (in sec) spent to complete the concentric phase of the CS-5 (21).

### Assessment and Definition of Other Variables

Demographic and other variables were collected by trained interviewers according to standard procedures. Age, sex, educational level, smoking and drinking status, place of residence, marital status, income, and number of chronic diseases were gathered using a standardized, structured interview questionnaire. For further analysis, age groups were classified into “40–44,” “45–49,” “50–54,” “55–59,” “60–64,” “65–69,” “70–74,” “75–79,” or “80+”; educational levels were classified into “no formal education,” “primary school,” or “middle school or above”; smoking status was classified as “current or ex-smoker” or not; drinking status was classified as “current or ex-drinker” or not; place of residence was classified as “rural village” or “urban community”; marital status was classified as “currently married” or not; income was classified as “≥ mean value” or not; and the number of chronic diseases was classified as “0” or “≥1.”

Height, body weight, and waist circumference were measured using a standard protocol. BMI was calculated as the weight in kilograms divided by the square of the height in meters (kg·m^−2^). Handgrip strength was measured using a hand-held dynamometer. Participants were tested by trained technicians under the same conditions. Participants were asked to perform two maximum-force trials for each hand. The greatest force was used as the final handgrip strength. Cognitive functioning (including orientation and attention, episodic memory, and visuo-construction) was assessed. The detailed information of cognitive functioning test can be found elsewhere (22).

### Statistical Analysis

Normality of the data was assessed using the Kolmogorov–Smirnov test and the appropriate parametric or non-parametric test was applied. The 5th, 25th, 50th, 75th, and 95th percentiles were chosen as age-specific and sex-specific percentiles of CS-5 time. Participant characteristics were stratified by sex. Continuous variables are presented as least-square means, standard deviations (SD), and 95% CIs; categorical variables are presented as counts and percentages. The cutoff values of CS-5 time were <1 SD by sex and age group. Significant differences between men and women group were analyzed by Student's *t*-test or the χ^2^-test. A multiple linear stepwise regression analysis (significance level for entry = 0.10, significance level to stay = 0.15) was developed to determine the extent to which CS-5 time was influenced by the participants' demographic (age, sex, marital status, place of residence, income, educational level, smoking and drinking status, and no. of chronic diseases) and anthropometric (height, weight, waist circumference, and handgrip strength) characteristics. All statistical analyses were performed using the Statistical Analysis System 9.4 edition for Windows (SAS Institute, Cary, NC, USA). All tests were two-tailed, and a *P* < 0.05 was considered significant.

## Results

### Study Population

We included 12,605 of 17,708 participants (71.8%) for reference values analyses, and 12,300 of 17,708 participants (69.5%) for risk factors analyses (Supplementary Figure 1). Among the 12,605 participants, their mean age was 58.3 years (SD, 9.39), and 6,641 (52.7%) of the participants were women. The mean CS-5 time was 10.13 s (SD, 3.32) in men and 11.03 s (SD, 3.54) in women aged 40+ years; 14.06 s (SD, 4.08) in men and 14.89 s (SD, 4.60) in women aged 80+ years.

### Reference Values for CS-5

**Figure 1:**
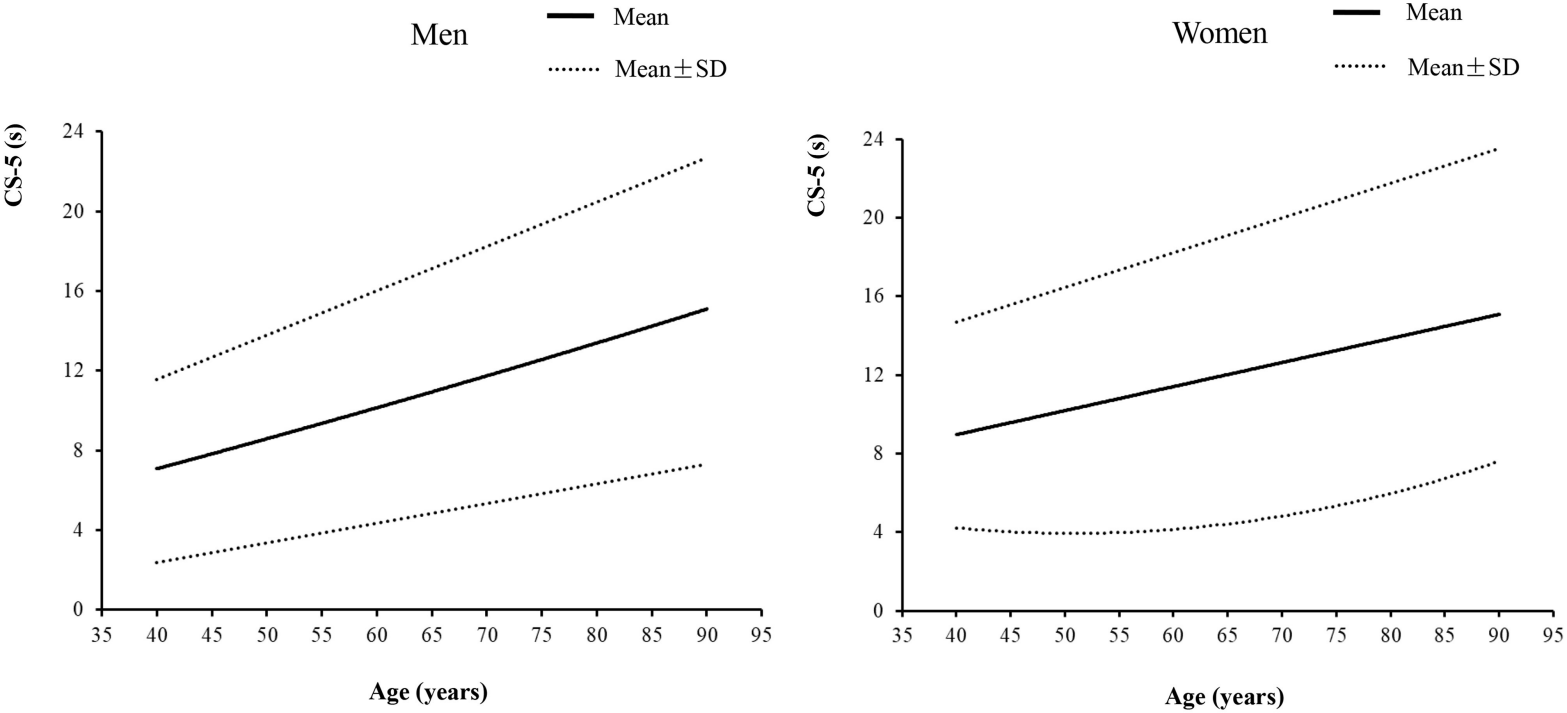
The five-repetition chair stand test times in middle-aged and elderly community-dwelling Chinese adults.

Table 1 and Figure 1 show smoothed age-specific and sex-specific percentiles of CS-5 time in men and women. The data show that men performed better at all ages than women. In men, the 50th percentile of CS-5 time ranged from 7.93 to 13.54 s and in women it was from 8.94 to 14.60 s. There was an increase in CS-5 time across the age range in both sexes. More details on the percentiles of CS-5 time are shown in Supplementary Tables 1, 2.

**Table 1:** Age-specific and sex-specific percentile of CS-5 time (in sec) in men and women (*n* = 12,605).

**Sex/age group**	***n***	**Mean** **(sec)**	**SD** **(sec)**	**P5** **(sec)**	**P25** **(sec)**	**P50** **(sec)**	**P75** **(sec)**	**P95** **(sec)**
**Men (*****n*** **= 5,964)**								
40–44	37	8.76	3.40	5.12	7.19	7.93	9.19	18.94
45–49	1,070	8.91	2.77	5.25	6.97	8.40	10.30	14.25
50–54	911	9.36	2.79	5.57	7.21	8.94	11.12	14.58
55–59	1,228	9.86	3.14	5.62	7.66	9.34	11.64	15.66
60–64	1,092	10.23	3.13	6.12	8.04	9.73	11.73	16.44
65–69	725	10.61	3.19	6.25	8.35	10.07	12.34	16.78
70–74	504	11.75	3.58	7.03	9.17	11.00	13.83	18.51
75–79	281	12.52	4.05	6.84	9.62	11.75	14.93	19.94
80+	116	14.06	4.08	7.68	11.19	13.54	16.59	22.66
**Women (*****n*** **= 6,641)**								
40–44	241	9.43	2.95	5.62	7.51	8.94	11.06	14.91
45–49	1,473	9.84	2.88	5.90	7.75	9.38	11.44	15.29
50–54	1,031	10.46	3.17	6.06	8.15	10.03	12.30	16.35
55–59	1,391	10.91	3.33	6.28	8.53	10.35	12.72	17.12
60–64	1,053	11.36	3.44	6.67	8.88	10.78	13.22	18.28
65–69	674	12.11	3.67	6.97	9.50	11.66	14.13	19.31
70–74	390	12.82	4.04	7.44	9.78	12.23	15.31	20.54
75–79	259	13.46	3.96	7.62	10.50	13.00	15.97	20.93
80+	129	14.89	4.60	7.07	11.38	14.60	18.22	22.75

*CS-5, 5-repetition chair stand test; P, percentile; SD, standard deviation*.

The cutoff values of CS-5 time using <1 SD by sex and age group are presented in Table 2. These cutoff points ranged from 5.36 to 9.98 s and from 6.48 to 10.29 s in men and women, respectively.

**Table 2:** CS-5 time (in sec) cutoff values using <1 SD by sex and age group (*n* = 12,605).

**Sex/age group**	**Cut point**
**Men (*****n*** **= 5,964)**	**Mean (sec)**
40–44	5.36
45–49	6.14
50–54	6.57
55–59	6.72
60–64	7.10
65–69	7.42
70–74	8.17
75–79	8.47
80+	9.98
**Women (*****n*** **= 6,641)**	
40–44	6.48
45–49	6.96
50–54	7.29
55–59	7.58
60–64	7.92
65–69	8.43
70–74	8.77
75–79	9.50
80+	10.29

*CS-5, 5-repetition chair stand test; SD, standard deviation*.

The characteristics of the participants for the risk factor analyses stratified by sex are presented in Table 3. The means (95% CIs) of CS-5 time (in sec) for men and women were 10.11 (10.02, 10.20) and 11.01(10.93, 11.09), respectively. The means (95% CIs) of mean velocity (in m·s^−1^) for men and women were 0.38 (0.38, 0.39) and 0.30 (0.29, 0.30), respectively. Females tended to be young, currently married, non-smokers, and non-drinkers; they also tended to have higher educational levels and BMI, but lower handgrip strength and annual income, and more chronic diseases (all *P* < 0.001).

**Table 3:** Participant characteristics according to sex (*n* = 12,300).

**Characteristics**	**Men (*n* = 5,829)**	**Women (*n* = 6,471)**	***P***
	**Mean (95% CI)**	**Mean (95% CI)**	
CS-5 time (s)	10.11 (10.02, 10.20)	11.01 (10.93, 11.09)	<0.001
Mean velocity (m·s^−1^)	0.38 (0.38, 0.39)	0.30 (0.29, 0.30)	<0.001
Age (years)	59.21 (58.97, 59.45)	57.43 (57.20, 57.65)	<0.001
BMI (kg·m^−2^)	22.94 (22.84, 23.04)	23.95 (23.86, 24.05)	<0.001
Waist (cm)	83.90 (83.58, 84.22)	84.28 (83.98, 84.60)	0.085
Handgrip strength (kg)	39.58 (39.33, 39.83)	27.18 (26.94, 27.41)	<0.001
Marital status (currently married, %)	5,281 (90.60)	5,561 (85.94)	<0.001
Residence in rural village (yes, %)	3,748 (64.30)	4,051 (62.60)	0.051
Income (≥ mean value)	1,381 (23.69)	654 (10.11)	<0.001
Educational level (%)			<0.001
No formal education	2,003 (34.36)	593 (9.16)	
Primary school	1,964 (33.69)	2,453 (37.91)	
Middle school or above	1,862 (31.94)	3,425 (52.93)	
Smoking status (current or ex-smoker, %)	4,369 (74.95)	504 (7.79)	<0.001
Drinking status (current or ex-drinker, %)	2,643 (45.34)	448 (6.92)	<0.001
No. of chronic diseases (≥1, %)	3,839 (65.86)	4,447 (68.72)	<0.001

*BMI, body mass index; CS-5, 5-repetition chair stand test; CI, confidence interval*.

### Reference Equations for CS-5

As shown in Table 4, multiple regression analysis was performed, where age (β = 0.092; *P* < 0.001), waist (β = 0.012; *P* < 0.001), BMI (β = 0.019; *P* = 0.051), place of residence (β = 0.516; *P* < 0.001), and no. of chronic diseases (β = 0.267; *P* < 0.001) were positively associated with CS-5 time and sex (β = −0.267; *P* < 0.001), handgrip strength (β = −0.049; *P* < 0.001), marital status (β = −0.237; *P* = 0.012), income (β = −0.321; *P* < 0.001), and drinking status (β = −0.286; *P* < 0.001) were negatively associated with CS-5 time in this population.

**Table 4:** Multiple liner stepwise regression of CS-5 time.

**Variable**	**β**	**SE**	***F*-value**	***P*-value**
Intercept	5.374	0.355	228.870	<0.001
Age (years)	0.092	0.004	642.530	<0.001
BMI (kg·m^−2^)	0.019	0.010	3.810	0.051
Waist (cm)	0.012	0.003	17.660	<0.001
Handgrip strength (kg)	−0.049	0.003	228.850	<0.001
Sex (male)^a^	−0.267	0.078	11.660	<0.001
Marital status (currently married)^b^	−0.237	0.094	6.350	0.012
Place of residence (rural village)^c^	0.516	0.061	70.470	<0.001
Income (≥ mean value)^d^	−0.321	0.083	14.780	<0.001
Drinking status (current or ex-drinker)^e^	−0.286	0.075	14.660	<0.001
No. of chronic diseases (≥1)^f^	0.267	0.063	18.240	<0.001

*BMI, body mass index; CS-5, 5-repetition chair stand test; SE, standard error*.

a *Male = 1; female = 0*.

b *Currently married = 1; divorced, windowed, or never married = 0*.

C *Rural village = 1; urban community = 0*.

d *Higher than or equal to mean value = 1; lower than mean value = 0*.

e *Current or ex-drinker = 1; non-drinker = 0*.

f *Have chronic diseases = 1; none = 0*.

## Discussion

In the present study, we established age- and sex-stratified reference values for the CS-5 from a large population of 12,605 Chinese community-dwelling adults, aged 40+ years. To the best of our knowledge, the present study is the first to define reference values for the CS-5 among middle aged and elderly Chinese. The present results suggest that the CS-5 time increased more with age in women than in men. Furthermore, our study identified parameters, such as waist circumference, handgrip strength, marital status, place of residence, income, and no. of chronic diseases, as independently associated with the CS-5 times.

Several normative data of the CS-5 for older adults from populations with different nationalities have been published in the last decade (11–14). However, reference values for the CS-5 have never been described in the Chinese adult population. The present findings are consistent with those of a previous study indicating that performance on the CS-5 increases with age across sexes. A previous study conducted in Colombia reported that the mean CS-5 times for men and women were 12.95 s (SD, 5.52) and 14.10 s (SD, 6.03), respectively (14). Furthermore, consistent with the present results, previous studies indicated that men did performed better on the CS-5 than women in the same age range. Ramírez-Vélez et al. suggested that performance on the CS-5 was different between men and women in the 60 to 69-, 70 to 79-, and 80+-year groups (all *P* < 0.001). The mean CS-5 times for Colombian older adults aged 80+ years were 15.94 s (SD, 6.14) for men and 16.00 s (SD, 7.02) for women (14). Another study conducted in Thailand suggested that performance on the CS-5 was different between men and women in the 70 to 79-year and 80+-year groups (all *P* < 0.001). The mean CS-5 times for Thai older adults aged 80+ years were 14.2 s (SD, 3.4) in men and 17.1 s (SD, 4.6) in women (11). Gender and age also affect performance on the 30-s chair stand test and 1-min chair stand test (15, 18).

Moreover, in the present study, the impact of health-related factors, such as higher BMI, lower handgrip strength, and residence in a rural village, on mobility also contributes to the observation. A previous study conducted on Filipinos reported that higher CS-5 time are related to lower BMI values [β = −0.020, *P* < 0.001; (13)]. Another study including 6,926 participants also reported that lower chair stand test performance was related to five BMI units [β = −0.283, *P* < 0.001; (18)]. Men and women in mid-life begin to show a decrease in muscle strength, such as handgrip strength, which is a good marker of physical performance (2). Stevens et al. reported that handgrip strength is associated with a 1% decrease in the CS-5 time [β = 0.99, *P* < 0.001; (23)]. Older people who live in Chinese rural villages have poorer health compared to their urban dwelling peers due to inadequate access to health care and resources (24). Consistent with the present results, Lunar et al. indicated that urban-dwellers perform better on the CS-5 than their rural dwelling counterparts (13).

Only three of eight previous studies provided chair heights in their reports (Supplementary Table 3). A standard armless chair is usually 43–47 cm in height (25). The participants for the CS-5 test sat on a standard armless chair with a seat height of 47 cm in the present study. Thaweewannakij et al. performed the CS-5 test using a 43 cm chair height. However, different seat heights may have increased the variability of the reference value results for the CS-5 (26). Height may be a risk factor for CS-5, and should be included in the reference equations for CS-5.

### Strengths and Limitations

The present study had several strengths. First, this study used a nationally representative sample that included 450 urban communities and rural villages within 28 provinces of China. Second, the present study is the largest and the first study to provide comprehensive reference data for the CS-5 in a Chinese adult population. The reference values were calculated for sex-specific, 5-year age spans to improve clinical applicability. Third, the analyses investigated the association between demographic and anthropometric factors and CS-5 times. Nevertheless, there were several limitations of this study. First, the study population was comprised of only Chinese adults, which limits generalizability of the results to other populations. Moreover, we excluded 4,802 participants who did not provide information on CS-5 time. Participants who had cognitive impairment could not follow an instruction and these participants would have been expected to perform poorly in CS-5. We found that there was no significant difference in total cognitive scores between participants who were included for reference values analyses and who were excluded due to missing data on CS-5 times (Supplementary Table 4). However, we also found that 12.1 and 45.9% participants were excluded due to missing data on cognitive scores in the included and excluded participants, respectively (Supplementary Table 4). Thus, participants who had missing data on CS-5 times tended to have missing data on cognitive scores, and participants who had missing data both on CS-5 times and cognitive scores would have been expected to perform poorly in CS-5. Furthermore, participants who were excluded due to missing data on CS-5 times were older than that of included participants. Thus, the presented results could be biased. Second, there could have been recall bias in this study. Third, it is impossible to infer causality due to the cross-sectional study design. Fourth, factors associated with chair stand are expected; what is really needed is the determinations of cut off values relating to adverse health outcomes, which require longitudinal and clinical data.

## Conclusion

The present study provides valid national reference standards for the CS-5 in Chinese middle-aged and elderly adults. The comprehensive normative values for CS-5 are essential to enable clinicians to better evaluate functional performance, determine an appropriate interventional strategy, and promote healthy aging of older adults.

## Data Availability Statement

The raw data supporting the conclusions of this article will be made available by the authors, without undue reservation.

## Author Contributions

S-YG and YX designed the study, formulated the clinical question, and analyzed the data. Y-HZ had full access to all data in the study and is responsible for data integrity and the accuracy of data analysis. All authors prepared, reviewed, revised, read, and approved the final manuscript.

## Conflict of Interest

The authors declare that the research was conducted in the absence of any commercial or financial relationships that could be construed as a potential conflict of interest.

## Supplementary Material

Supplementary Figure 1 Flow chart of the selection of study participants.

Supplementary Table 1 CS-5 time (in sec) percentiles for men, by age.

Supplementary Table 2 CS-5 time (in sec) percentiles for women, by age.

Supplementary Table 3 Characteristics of studies of reference values for chair stand test reported in the past decade.

Supplementary Table 4 Characteristics of participants according to exclusion of CS-5.

## References

[1] Zeng Y. Towards deeper research and better policy for healthy aging –using the unique data of Chinese longitudinal healthy longevity survey. China Economic J. (2012) 5:131–49. 10.1080/17538963.2013.764677 24443653 PMC3893304

[2] Landi F Calvani R Tosato M Martone AM Fusco D Sisto A. Age-related variations of muscle mass, strength, and physical performance in community-dwellers: results from the Milan EXPO survey. J Am Med Direct Assoc. (2017) 18:88.e17–88.e24. 10.1016/j.jamda.2016.10.007 27914849

[3] García-Hermoso A Cavero-Redondo I Ramírez-Vélez R Ruiz JR Ortega FB Lee DC. Muscular strength as a predictor of all-cause mortality in an apparently healthy population: a systematic review and meta-analysis of data from approximately 2 million men and women. Archiv Phys Med Rehabil. (2018) 99:2100–13.e5. 10.1016/j.apmr.2018.01.008 29425700

[4] Li R Xia J Zhang XI Gathirua-Mwangi WG Guo J Li Y. Associations of muscle mass and strength with all-cause mortality among US older adults. Med Sci Sports Exerc. (2018) 50:458–67. 10.1249/MSS.0000000000001448 28991040 PMC5820209

[5] Mehmet H Yang AWH Robinson SR. What is the optimal chair stand test protocol for older adults? A systematic review. Disabil Rehabil. (2020) 42:2828–35. 10.1080/09638288.2019.1575922 30907166

[6] Pavasini R Guralnik J Brown JC di Bari M Cesari M Landi F. Short physical performance battery and all-cause mortality: systematic review and meta-analysis. BMC Med. (2016) 14:215. 10.1186/s12916-016-0763-7 28003033 PMC5178082

[7] Jones SE Kon SSC Canavan JL Patel MS Clark AL Nolan CM. The five-repetition sit-to-stand test as a functional outcome measure in COPD. Thorax. (2013) 68:1015–20. 10.1136/thoraxjnl-2013-203576 23783372

[8] Ng SS Ng HH Chan KM Lai JC To AK Yeung CW. Reliability of the 12-step ascend and descend test and its correlation with motor function in people with chronic stroke. J Rehabil Med. (2013) 45:123–9. 10.2340/16501977-1086 23307269

[9] Nero H Dahlberg J Dahlberg LE. A 6-week web-based osteoarthritis treatment program: observational quasi-experimental study. J Med Int Res. (2017) 19:e422. 10.2196/jmir.9255 29254906 PMC5748477

[10] Sitjà-Rabert M Martínez-Zapata MJ Fort Vanmeerhaeghe A Rey Abella F Romero-Rodríguez D Bonfill X. Effects of a whole body vibration (WBV) exercise intervention for institutionalized older people: a randomized, multicentre, parallel, clinical trial. J Am Med Direct Assoc. (2015) 16:125–31. 10.1016/j.jamda.2014.07.018 25282631

[11] Thaweewannakij T Wilaichit S Chuchot R Yuenyong Y Saengsuwan J Siritaratiwat W. Reference values of physical performance in Thai elderly people who are functioning well and dwelling in the community. Phys Ther. (2013) 93:1312–20. 10.2522/ptj.20120411 23620530

[12] Bergland A Strand BH. Norwegian reference values for the Short Physical Performance Battery (SPPB): the Tromsø Study. BMC Geriatr. (2019) 19:216. 10.1186/s12877-019-1234-8 31395008 PMC6686475

[13] Lunar FR Marquez JP Quianzon FK Policarpio BJ Santelices LA Velasco MK. Mobility performance among community-dwelling older Filipinos who lived in urban and rural settings: a preliminary study. Hong Kong Physiother J. (2019) 39:91–9. 10.1142/S1013702519500082 31889760 PMC6900329

[14] Ramírez-Vélez R Pérez-Sousa MA Venegas-Sanabria LC Cano-Gutierrez CA Hernández-Quiñonez PA Rincón-Pabón D. Normative values for the Short Physical Performance Battery (SPPB) and their association with anthropometric variables in older colombian adults. The SABE study, 2015. Front Med. (2020) 7:52. 10.3389/fmed.2020.00052 PMC7044127 32154258

[15] Tveter AT Dagfinrud H Moseng T Holm I. Health-related physical fitness measures: reference values and reference equations for use in clinical practice. Archiv Phys Med Rehabil. (2014) 95:1366–73. 10.1016/j.apmr.2014.02.016 24607837

[16] Gunasekaran V Banerjee J Dwivedi SN Upadhyay AD Chatterjee P Dey AB. Normal gait speed, grip strength and thirty seconds chair stand test among older Indians. Archiv Gerontol Geriatr. (2016) 67:171–8. 10.1016/j.archger.2016.08.003 27552583

[17] McKay MJ Baldwin JN Ferreira P Simic M Vanicek N Burns J. Reference values for developing responsive functional outcome measures across the lifespan. Neurology. (2017) 88:1512–9. 10.1212/WNL.0000000000003847 28330961

[18] Strassmann A Steurer-Stey C Lana KD Zoller M Turk AJ Suter P. Population-based reference values for the 1-min sit-to-stand test. Int J Public Health. (2013) 58:949–53. 10.1007/s00038-013-0504-z 23974352

[19] Nakazono T Kamide N Ando M. The reference values for the chair stand test in healthy Japanese older people: determination by meta-analysis. J Phys Ther Sci. (2014) 26:1729–31. 10.1589/jpts.26.1729 25435687 PMC4242942

[20] Zhao Y Hu Y Smith JP Strauss J Yang G. Cohort profile: the China Health and Retirement Longitudinal Study (CHARLS). Int J Epidemiol. (2014) 43:61–8. 10.1093/ije/dys203 23243115 PMC3937970

[21] Alcazar J Losa-Reyna J Rodriguez-Lopez C Alfaro-Acha A Rodriguez-Mañas L Ara I. The sit-to-stand muscle power test: an easy, inexpensive and portable procedure to assess muscle power in older people. Exp Gerontol. (2018) 112:38–43. 10.1016/j.exger.2018.08.006 30179662

[22] Cao L Zhao Z Ji C Xia Y. Association between solid fuel use and cognitive impairment: a cross-sectional and follow-up study in a middle-aged and older Chinese population. Environ Int. (2021) 146:106251. 10.1016/j.envint.2020.106251 33248346

[23] StevensPJSyddallHEPatelHPMartinHJCooperCAihie SayerA. Is grip strength a good marker of physical performance among community-dwelling older people? J Nutr Health Aging. (2012) 16:769–74. 10.1007/s12603-012-0388-223131819

[24] Zhang X Dupre ME Qiu L Zhou W Zhao Y Gu D. Urban-rural differences in the association between access to healthcare and health outcomes among older adults in China. BMC Geriatr. (2017) 17:151. 10.1186/s12877-017-0538-9 28724355 PMC5516359

[25] Paul SS Canning CG. Five-repetition sit-to-stand. J Physiother. (2014) 60:168. 10.1016/j.jphys.2014.06.002 25066936

[26] Bohannon RW. Reference values for the five-repetition sit-to-stand test: a descriptive meta-analysis of data from elders. Percept Motor Skills. (2006) 103:215–22. 10.2466/pms.103.1.215-222 17037663

