# Supplementary figures and images for: Reference Values for Five-Repetition Chair Stand Test Among Middle-Aged and Elderly Community-Dwelling Chinese Adults
Source: Front Med (Lausanne). 2021 Apr 21;8:659107. doi: 10.3389/fmed.2021.659107 (PMC8096929; doi:10.3389/fmed.2021.659107)

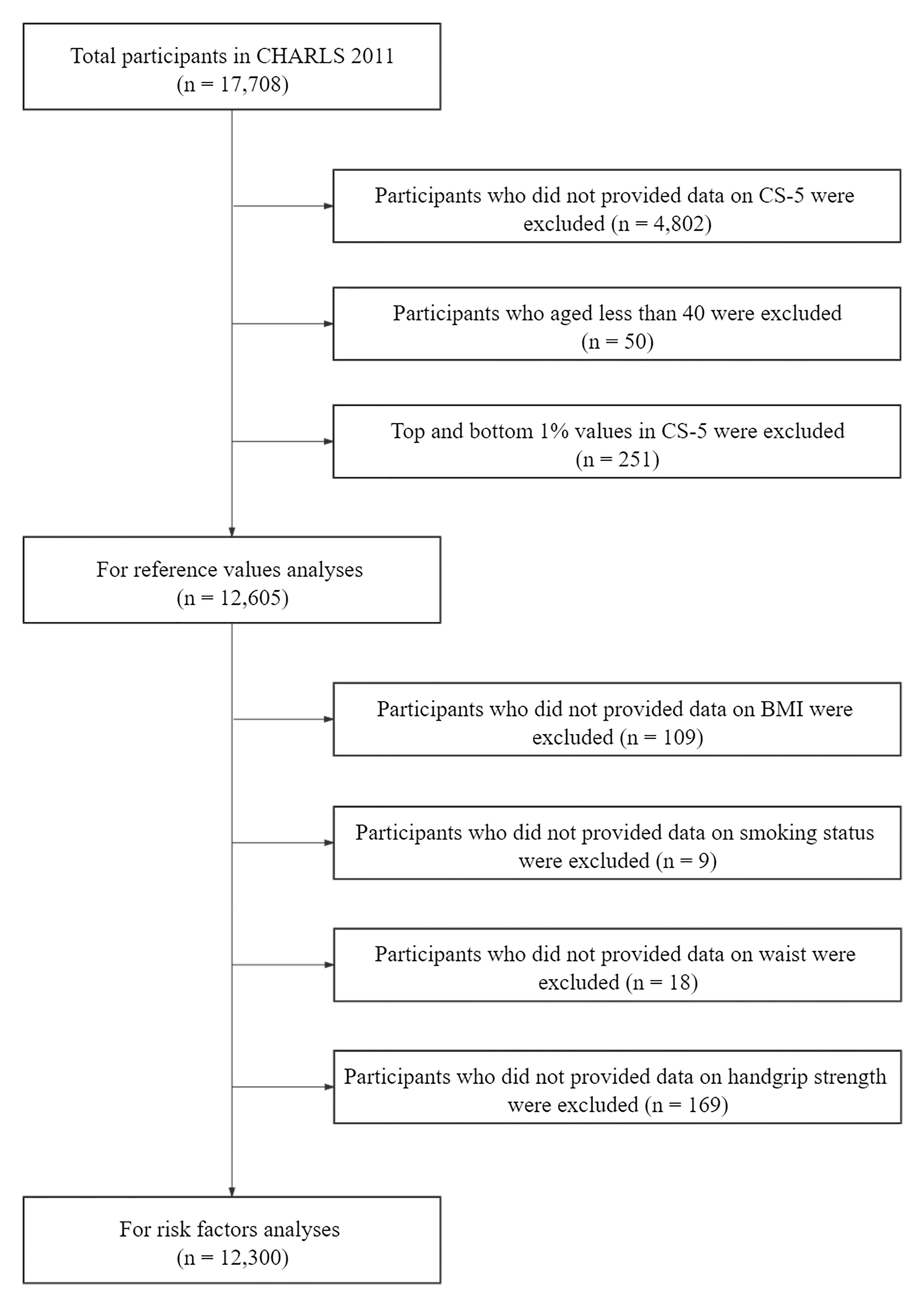

Supplement: Supplementary Figure 1 — Flow chart of the selection of study participants. [file Image_1.TIF]
