# Supplementary material for: Reference Values for Five-Repetition Chair Stand Test Among Middle-Aged and Elderly Community-Dwelling Chinese Adults
Source: Front Med (Lausanne). 2021 Apr 21;8:659107. doi: 10.3389/fmed.2021.659107 (PMC8096929; doi:10.3389/fmed.2021.659107)
Supplement: Supplementary Table 1 — CS-5 time (in sec) percentiles for men, by age. [file Table_1.doc]

| Table S1. CS-5 time (in sec) percentiles for men, by age | | | | | | | | |
| --- | --- | --- | --- | --- | --- | --- | --- | --- |
| Age (years) | n | Mean | SD | P5 | P25 | P50 | P75 | P95 |
| 41 | 2 | 8.47 | 0.71 | 7.97 | 7.97 | 8.47 | 8.97 | 8.97 |
| 42 | 1 | 7.40 | N/A | 7.40 | 7.40 | 7.40 | 7.40 | 7.40 |
| 43 | 8 | 9.73 | 2.74 | 7.50 | 7.70 | 8.87 | 10.84 | 15.54 |
| 44 | 26 | 8.53 | 3.76 | 5.12 | 6.08 | 7.84 | 8.95 | 18.94 |
| 45 | 165 | 8.74 | 2.70 | 5.12 | 6.93 | 8.18 | 10.06 | 14.37 |
| 46 | 222 | 8.85 | 2.65 | 5.50 | 6.87 | 8.24 | 10.31 | 13.67 |
| 47 | 210 | 8.98 | 2.64 | 5.47 | 7.15 | 8.47 | 10.37 | 14.03 |
| 48 | 274 | 9.04 | 2.82 | 5.16 | 7.00 | 8.68 | 10.67 | 14.60 |
| 49 | 199 | 8.86 | 3.02 | 5.23 | 6.77 | 8.43 | 9.97 | 15.74 |
| 50 | 116 | 8.72 | 2.84 | 5.31 | 6.60 | 8.08 | 10.31 | 14.19 |
| 51 | 174 | 8.97 | 2.55 | 5.37 | 7.03 | 8.81 | 10.41 | 13.72 |
| 52 | 133 | 9.95 | 2.66 | 6.25 | 7.93 | 9.68 | 11.69 | 14.55 |
| 53 | 241 | 9.34 | 2.76 | 5.31 | 7.50 | 8.97 | 11.10 | 14.68 |
| 54 | 247 | 9.65 | 2.94 | 6.00 | 7.41 | 9.06 | 11.32 | 15.12 |
| 55 | 225 | 9.97 | 3.08 | 5.65 | 7.84 | 9.34 | 11.84 | 16.03 |
| 56 | 258 | 9.74 | 3.17 | 5.60 | 7.66 | 9.13 | 11.37 | 16.59 |
| 57 | 235 | 9.85 | 3.27 | 5.66 | 7.56 | 9.40 | 11.65 | 16.00 |
| 58 | 255 | 9.70 | 3.08 | 5.53 | 7.47 | 9.19 | 11.60 | 15.00 |
| 59 | 255 | 10.04 | 3.09 | 5.78 | 7.98 | 9.72 | 11.78 | 15.91 |
| 60 | 240 | 10.31 | 3.29 | 6.13 | 8.00 | 9.63 | 11.96 | 16.84 |
| 61 | 213 | 10.08 | 3.16 | 6.09 | 7.69 | 9.69 | 11.72 | 15.88 |
| 62 | 241 | 9.98 | 2.98 | 5.81 | 8.03 | 9.53 | 11.59 | 15.60 |
| 63 | 190 | 10.59 | 3.35 | 6.47 | 8.15 | 9.97 | 12.75 | 17.12 |
| 64 | 208 | 10.26 | 2.87 | 6.56 | 8.44 | 10.12 | 11.21 | 15.69 |
| 65 | 158 | 10.33 | 3.00 | 6.18 | 8.28 | 9.76 | 12.22 | 16.41 |
| 66 | 147 | 10.46 | 3.10 | 6.31 | 8.06 | 9.91 | 11.81 | 16.78 |
| 67 | 154 | 10.65 | 3.27 | 6.60 | 8.28 | 10.07 | 12.22 | 17.56 |
| 68 | 135 | 10.52 | 3.13 | 5.85 | 8.34 | 10.12 | 12.34 | 16.34 |
| 69 | 131 | 11.17 | 3.43 | 6.25 | 8.87 | 10.59 | 13.09 | 17.00 |
| 70 | 115 | 11.19 | 3.54 | 6.43 | 8.93 | 10.65 | 13.30 | 17.30 |
| 71 | 101 | 11.38 | 3.56 | 7.34 | 9.21 | 10.57 | 12.81 | 19.50 |
| 72 | 90 | 11.60 | 3.52 | 7.53 | 9.15 | 10.61 | 13.69 | 18.09 |
| 73 | 108 | 12.25 | 3.28 | 7.54 | 9.69 | 11.71 | 14.58 | 18.13 |
| 74 | 90 | 12.43 | 3.93 | 6.90 | 10.00 | 11.56 | 14.81 | 20.72 |
| 75 | 89 | 12.52 | 4.01 | 6.84 | 9.86 | 12.00 | 14.75 | 21.47 |
| 76 | 54 | 12.38 | 4.48 | 6.84 | 9.22 | 11.01 | 14.19 | 21.81 |
| 77 | 55 | 12.05 | 3.92 | 6.28 | 8.95 | 11.67 | 15.38 | 18.43 |
| 78 | 46 | 13.20 | 4.27 | 6.41 | 10.12 | 12.46 | 15.34 | 20.00 |
| 79 | 37 | 12.55 | 3.43 | 7.47 | 9.84 | 13.09 | 15.00 | 18.22 |
| 80 | 29 | 13.18 | 3.78 | 7.62 | 11.09 | 12.68 | 15.47 | 18.81 |
| Age (years) | n | Mean | SD | P5 | P25 | P50 | P75 | P95 |
| 81 | 20 | 13.79 | 4.00 | 9.69 | 10.56 | 13.21 | 15.41 | 23.22 |
| 82 | 17 | 13.82 | 3.37 | 9.57 | 11.19 | 12.34 | 16.07 | 19.90 |
| 83 | 14 | 15.26 | 5.42 | 6.22 | 10.82 | 16.03 | 19.06 | 23.84 |
| 84 | 8 | 12.99 | 3.41 | 8.44 | 10.02 | 13.38 | 15.24 | 18.22 |
| 85 | 10 | 15.99 | 4.04 | 11.19 | 11.41 | 15.97 | 19.22 | 22.69 |
| 86 | 8 | 14.16 | 4.51 | 6.69 | 11.99 | 13.08 | 17.70 | 21.09 |
| 87 | 3 | 18.57 | 3.55 | 16.21 | 16.21 | 16.85 | 22.66 | 22.66 |
| 88 | 1 | 16.43 | N/A | 16.43 | 16.43 | 16.43 | 16.43 | 16.43 |
| 89 | 3 | 15.70 | 1.42 | 14.16 | 14.16 | 15.97 | 16.97 | 16.97 |
| 90 | 2 | 10.25 | 2.83 | 8.25 | 8.25 | 10.25 | 12.25 | 12.25 |
| 93 | 1 | 7.46 | N/A | 7.46 | 7.46 | 7.46 | 7.46 | 7.46 |
| CS-5, 5-repetition chair stand test; M, median; N/A, not applicable; P, percentile; SD, standard deviation. | | | | | | | | |

| Table S2. CS-5 time (in sec) percentiles for women, by age | | | | | | | | |
| --- | --- | --- | --- | --- | --- | --- | --- | --- |
| Age (years) | n | Mean | SD | P5 | P25 | P50 | P75 | P95 |
| 40 | 22 | 8.88 | 2.23 | 4.89 | 8.19 | 9.46 | 10.37 | 11.87 |
| 41 | 19 | 9.16 | 2.87 | 5.41 | 7.00 | 8.22 | 10.44 | 16.84 |
| 42 | 40 | 9.60 | 2.97 | 5.11 | 7.72 | 9.43 | 10.64 | 15.21 |
| 43 | 67 | 9.72 | 2.80 | 6.16 | 7.68 | 9.00 | 11.53 | 14.94 |
| 44 | 93 | 9.32 | 3.22 | 5.72 | 7.10 | 8.47 | 11.19 | 15.43 |
| 45 | 257 | 9.51 | 2.49 | 5.90 | 7.51 | 9.27 | 11.28 | 14.01 |
| 46 | 296 | 9.84 | 3.06 | 5.78 | 7.55 | 9.43 | 11.61 | 15.29 |
| 47 | 271 | 9.97 | 2.84 | 6.09 | 8.00 | 9.31 | 11.54 | 15.82 |
| 48 | 394 | 9.85 | 2.85 | 5.82 | 7.85 | 9.47 | 11.50 | 15.57 |
| 49 | 255 | 10.02 | 3.10 | 5.97 | 7.81 | 9.65 | 11.41 | 15.50 |
| 50 | 152 | 9.97 | 2.89 | 5.87 | 7.96 | 9.35 | 11.69 | 15.69 |
| 51 | 200 | 10.34 | 3.19 | 6.29 | 7.97 | 10.11 | 11.92 | 16.25 |
| 52 | 174 | 10.93 | 3.28 | 6.28 | 8.59 | 10.48 | 12.91 | 16.70 |
| 53 | 235 | 10.43 | 3.14 | 6.00 | 8.09 | 9.97 | 12.41 | 16.06 |
| 54 | 270 | 10.55 | 3.22 | 6.13 | 8.23 | 10.00 | 12.30 | 16.38 |
| 55 | 236 | 10.57 | 3.29 | 5.75 | 8.22 | 10.03 | 12.53 | 16.25 |
| 56 | 298 | 10.68 | 3.25 | 6.12 | 8.29 | 10.30 | 12.50 | 16.75 |
| 57 | 290 | 11.23 | 3.46 | 6.16 | 8.60 | 10.72 | 13.56 | 17.44 |
| 58 | 254 | 11.26 | 3.51 | 6.38 | 8.72 | 10.89 | 13.12 | 18.32 |
| 59 | 313 | 10.78 | 3.13 | 6.72 | 8.69 | 10.34 | 12.44 | 16.87 |
| 60 | 244 | 11.35 | 3.62 | 6.44 | 8.56 | 10.87 | 13.18 | 18.45 |
| 61 | 222 | 11.08 | 3.12 | 6.75 | 8.88 | 10.52 | 13.15 | 16.72 |
| 62 | 215 | 11.22 | 3.19 | 6.89 | 8.94 | 10.72 | 13.04 | 16.71 |
| 63 | 176 | 11.51 | 3.70 | 6.53 | 8.69 | 11.02 | 13.27 | 19.07 |
| 64 | 196 | 11.70 | 3.55 | 7.06 | 9.16 | 11.09 | 13.66 | 18.35 |
| 65 | 154 | 12.23 | 3.94 | 6.78 | 9.30 | 11.83 | 14.69 | 20.65 |
| 66 | 153 | 11.80 | 3.45 | 7.03 | 9.41 | 11.23 | 13.25 | 18.75 |
| 67 | 135 | 12.04 | 3.38 | 7.21 | 9.53 | 11.57 | 13.78 | 18.18 |
| 68 | 108 | 12.52 | 4.08 | 6.88 | 9.51 | 12.11 | 14.91 | 20.47 |
| 69 | 124 | 12.05 | 3.56 | 6.81 | 9.78 | 12.05 | 13.83 | 18.46 |
| 70 | 114 | 12.22 | 3.87 | 6.87 | 9.78 | 11.44 | 14.44 | 20.54 |
| 71 | 77 | 12.85 | 4.21 | 7.56 | 9.35 | 12.00 | 14.93 | 22.22 |
| 72 | 69 | 12.94 | 3.91 | 7.09 | 9.68 | 12.35 | 15.41 | 19.72 |
| 73 | 81 | 12.58 | 3.78 | 7.83 | 9.68 | 12.52 | 14.50 | 18.40 |
| 74 | 49 | 14.40 | 4.50 | 8.31 | 10.66 | 13.78 | 17.34 | 22.91 |
| 75 | 76 | 12.99 | 3.57 | 7.68 | 10.31 | 12.77 | 15.40 | 19.13 |
| 76 | 62 | 14.47 | 3.77 | 9.44 | 11.41 | 13.57 | 17.00 | 20.93 |
| 77 | 37 | 12.68 | 3.94 | 6.94 | 9.44 | 11.68 | 15.06 | 22.13 |
| 78 | 50 | 13.36 | 4.52 | 6.50 | 9.97 | 13.16 | 16.45 | 22.28 |
| Age (years) | n | Mean | SD | P5 | P25 | P50 | P75 | P95 |
| 79 | 34 | 13.67 | 4.10 | 7.62 | 10.81 | 12.88 | 17.44 | 21.44 |
| 80 | 24 | 14.28 | 4.34 | 8.22 | 10.69 | 14.19 | 18.09 | 20.35 |
| 81 | 23 | 16.22 | 4.56 | 9.85 | 11.47 | 16.59 | 20.59 | 22.75 |
| 82 | 24 | 14.79 | 5.29 | 5.93 | 10.05 | 14.03 | 19.61 | 22.35 |
| 83 | 15 | 13.63 | 5.03 | 5.37 | 9.27 | 13.07 | 18.22 | 23.50 |
| 84 | 11 | 14.03 | 4.50 | 5.00 | 10.28 | 14.60 | 17.28 | 20.91 |
| 85 | 7 | 15.82 | 5.43 | 8.79 | 10.21 | 16.00 | 21.19 | 23.87 |
| 86 | 3 | 15.31 | 3.55 | 11.91 | 11.91 | 15.03 | 19.00 | 19.00 |
| 87 | 6 | 16.34 | 4.65 | 10.45 | 13.85 | 15.88 | 17.78 | 24.22 |
| 88 | 5 | 12.15 | 2.87 | 8.28 | 10.66 | 12.44 | 13.45 | 15.90 |
| 89 | 2 | 14.20 | 1.44 | 13.18 | 13.18 | 14.20 | 15.22 | 15.22 |
| 90 | 3 | 14.00 | 2.37 | 11.38 | 11.38 | 14.63 | 16.00 | 16.00 |
| 91 | 4 | 18.27 | 4.58 | 13.38 | 15.38 | 17.62 | 21.16 | 24.44 |
| 94 | 1 | 16.21 | N/A | 16.21 | 16.21 | 16.21 | 16.21 | 16.21 |
| 95 | 1 | 16.01 | N/A | 16.01 | 16.01 | 16.01 | 16.01 | 16.01 |
| CS-5, 5-repetition chair stand test; M, median; N/A, not applicable; P, percentile; SD, standard deviation. | | | | | | | | |

| Table S3. Characteristics of studies of reference values for chair stand test reported in the past decade | | | | | | | | |
| --- | --- | --- | --- | --- | --- | --- | --- | --- |
| **First author, year** | **Age** | **Population** | **Setting** | **Sample size  (female, %)** | **Type of chair stand test** | **Characteristic of chair** | **Arm use permitted** | **Reference values presented** |
| Ramírez-Vélez R et al.^[1]^, 2020 | ≧ 60 years | Older Colombian adults | Urban and rural households | 4,211 (57.3%) | Five times sit-to-stand test | N/A | N/A | Crude mean values and SD, normative centiles (3rd, 10th, 25th, 50th, 75th, 90th, and 97th) stratified by sex and age. |
| Lunar FR et al.^[2]^, 2019 | ≧ 60 years | Filipino older adults | Community-dwelling | 150 (72.0%) | Five Times Sit to Stand Test | N/A | N/A | Mean values, SD, and 95% CI for the total group and stratified by place of residence. |
| Bergland A et al.^[3]^, 2019 | ≧ 40 years | Norwegian adults | Community-dwelling | 7,474 (53.2%) | 5-repeated chair sit-to-stand test | N/A | No | Percentiles (5th, 10th, 25th, 50th, 75th, 90th, and 95th percentiles), crude mean values and SD stratified by sex and age group. |
| McKay MJ et al.^[4]^, 2017 | 3–101 years | Children, adolescents, adults, older adults in Australia | The healthy ‘normal’ population across the lifespan | 1000 (50.0%) | 30-second chair stand test | N/A | N/A | Mean values, SD, 95th and 99th percentiles stratified by age per decade and sex. |
| Gunasekaran V et al.^[5]^, 2016 | ≧ 60 years | Older Indians | Outpatient department and ward of Geriatric Medicine department | 723 (31.7%) | Thirty seconds chair stand test | N/A | No | Median and interquartile range (25th and 75th quartiles) stratified by sex and 5-year age groups. |
| **First author, year** | **Age** | **Population** | **Setting** | **Sample size  (female, %)** | **Type of chair stand test** | **Characteristic of chair** | **Arm use permitted** | **Reference values presented** |
| Tveter AT et al.^[6]^, 2014 | 18–90 years | Older adults in the southeast part of Norway | Work sites, schools, community centers | 370 (51.9%) | 30-second sit-to-stand test | Height: 44–45cm | N/A | Median and interquartile range (25th and 75th quartiles) for the total group and stratified by separate sex and 10-year age groups. |
| Strassmann A et al.^[7]^, 2013 | 20–79 years | Switzerland adult population | Population-based | 6,926 (52.3%) | 1-min Sit-To-Stand Test | Height: 46 cm | No | Percentiles (2.5, 25, 50, 75 and 97.5th) stratified by sex and 5-year age groups. |
| Thaweewannakij T et al.^[8]^, 2013 | ≧ 60 years | Thai elderly people | Functioning well and dwelling in the community | 1,030 (68.9%) | Five Times Sit-to-Stand Test | Height: 43 cm | No | Mean values, SD, and 95% CI stratified by decade of age and sex. |
| N/A, not applicable; SD, standard deviation; CI, confidence interval. | | | | | | | | |

| Table S4. Characteristics of participants according to exclusion of CS-5 | | | |
| --- | --- | --- | --- |
|  | **Study population**  **(n = 11,084)** | **Participants with missing data on CS-5 time (n = 2,596)** | ***P* value ^*^** |
| No. of participants in the main analyses | 12,605 | 4,802 |  |
| Participants excluded due to missing data on cognitive score (%) | 12.1% | 45.9% |  |
| Age (years) | 57.98 (57.80, 58.15) ^†^ | 59.45 (59.09, 59.82) | <0.0001 |
| Sex (male, %) | 48.44 | 47.11 | 0.22 |
| Total cognitive score | 12.98 (12.89, 13.08) | 13.17 (12.97, 13.37) | 0.09 |
| Orientation and attention score | 5.15 (5.10, 5.19) | 5.25 (5.15, 5.34) | 0.06 |
| Episodic memory sscore | 7.16 (7.10, 7.23) | 7.28 (7.14, 7.41) | 0.13 |
| Visuo-construction score | 0.67 (0.66, 0.68) | 0.65 (0.63, 0.67) | 0.02 |

CS-5, 5-repetition chair stand test;

* Continuous and categorical variable are compared using analyses of variance and chi-square tests, respectively.

† Continuous variables are presented as mean (95% confidence interval) (all such values).

**References**

1. Ramírez-Vélez R, Pérez-Sousa MA, Venegas-Sanabria LC, Cano-Gutierrez CA, Hernández-Quiñonez PA, Rincón-Pabón D, García-Hermoso A, Zambom-Ferraresi F, Sáez de Asteasu ML, Izquierdo M. Normative Values for the Short Physical Performance Battery (SPPB) and Their Association With Anthropometric Variables in Older Colombian Adults. The SABE Study, 2015. Front Med (Lausanne) 2020, **7:** 52.

2. Lunar FR, Marquez JP, Quianzon FK, Policarpio BJ, Santelices LA, Velasco MK, Quinto RJ, Gorgon EJ. Mobility performance among community-dwelling older Filipinos who lived in urban and rural settings: A preliminary study. Hong Kong Physiother J 2019, **39**(2)**:** 91-99.

3. Bergland A, Strand BH. Norwegian reference values for the Short Physical Performance Battery (SPPB): the Tromsø Study. BMC Geriatr 2019, **19**(1)**:** 216.

4. McKay MJ, Baldwin JN, Ferreira P, Simic M, Vanicek N, Burns J. Reference values for developing responsive functional outcome measures across the lifespan. Neurology 2017, **88**(16)**:** 1512-1519.

5. Gunasekaran V, Banerjee J, Dwivedi SN, Upadhyay AD, Chatterjee P, Dey AB. Normal gait speed, grip strength and thirty seconds chair stand test among older Indians. Arch Gerontol Geriatr 2016, **67:** 171-178.

6. Tveter AT, Dagfinrud H, Moseng T, Holm I. Health-related physical fitness measures: reference values and reference equations for use in clinical practice. Arch Phys Med Rehabil 2014, **95**(7)**:** 1366-1373.

7. Strassmann A, Steurer-Stey C, Lana KD, Zoller M, Turk AJ, Suter P, Puhan MA. Population-based reference values for the 1-min sit-to-stand test. Int J Public Health 2013, **58**(6)**:** 949-953.

8. Thaweewannakij T, Wilaichit S, Chuchot R, Yuenyong Y, Saengsuwan J, Siritaratiwat W, Amatachaya S. Reference values of physical performance in Thai elderly people who are functioning well and dwelling in the community. Phys Ther 2013, **93**(10)**:** 1312-1320.
